# Should Minimally Invasive Surfactant Therapy be a Must in Neonatal Intensive Care Units? Pilot Report of Initial Cases in Dubai

**DOI:** 10.7759/cureus.3495

**Published:** 2018-10-25

**Authors:** Karthikeyan Gengaimuthu

**Affiliations:** 1 Neonatology and Pediatrics, International Modern Hospital, Dubai, ARE

**Keywords:** neonate, surfactant therapy, respiratory distress syndrome, minimally invasive surfactant therapy, non-invasive ventilation

## Abstract

Minimally invasive surfactant therapy (MIST) in neonates without intubation has in recent times become an accepted standard in the neonatal intensive care units (NICU) especially in Europe. We have adapted this novel technique in our clinical practice in the neonatal intensive care units in Dubai since 2018. Herein we report the successful outcome of implementation of this technique in three of our neonates of gestation/weight, 28/1.03 kg, 34/2.0 kg and 28/1.18 kg respectively in our NICU in Dubai, United Arab Emirates.

## Introduction

Non-invasive ventilation (NIV, that is avoiding intubation and consequent barotraumas) is the current standard of care in managing respiratory distress in very low birth weight neonates (babies less than 1500 g birth weight) [1-4]. The currently approved modes of non-invasive respiratory support are CPAP (continuous positive airway pressure) by nasal or nasopharyngeal route, HFNC (high-flow nasal cannula) and assisted CPAP techniques like non-synchronized or synchronized ventilator delivered breaths via nasal devices and even nasal CPAP + high frequency oscillatory ventilation [5-7]. Meta-analysis has shown that NIV significantly improves the morbidity and/or mortality due to chronic lung disease [7].

When the baby does need escalation of treatment despite being on CPAP, surfactant administration is needed. Traditionally surfactant was delivered intratracheally and baby needed to be ventilated. INSURE (INtubate SUrfactant Extubate) technique was evolved in Scandinavian countries to minimize the duration of intubation and ventilation [8-10]. But even INSURE fails to ensure airway injury and hence alternative minimally invasive ways of administration are being practiced and studied. In minimally invasive surfactant therapy (MIST), we continue the baby on CPAP and administer the surfactant using a thin catheter threaded into the trachea. 4 or 5 F infant feeding tubes are commonly used and guided using a Magill forceps. Semi rigid angiocaths that can be inserted without the aid of Magill forceps are being studied by another study group [6]. Follow-up studies and a recent meta-analysis have proven the superiority of MIST over conventional INSURE [11].

We in the International Modern Hospital, Dubai have mastered this MIST technique and have been successfully administering surfactant (both survanta and curosurf) to our babies since early 2018 and herein we present the initial three babies who received surfactant by MIST.

## Case presentation

Case 1

This 28 weeks/1.03 kg male neonate was delivered by emergency caesarian section to a high-risk mother with pre-eclampsia in May 2018. Baby needed bag and mask ventilation for three minutes due to poor respiratory efforts. From the 10 minutes of life the baby developed grunting and hence shifted to neonatal intensive care unit (NICU) on oxygen and started on nasal CPAP pressure 5 cm and FiO2: 0.28. He had apnea at four hours of life (probable antenatal magnesium sulphate effect) that was reverted with intravenous calcium and caffeine and the baby was continued on nasal CPAP. Umbilical vein was catheterized and preterm care initiated. X-ray was suggestive of hyaline membrane disease with streaky opacities and air bronchograms bilaterally.

The respiratory distress worsened and baby needed surfactant therapy by 16 hours of life. Under sterile precautions survanta (bovine surfactant) 5 ml was administered via a 5 F infant feeding tube guided into the trachea using Magill forceps while continuing on nasal CPAP (MIST by Calogne alias Magill forceps technique). The baby withstood the process well and X-ray showed clearing of the lungs 11 hours after the MIST therapy and baby was weaned off CPAP support at 28 hours after the MIST to incubator oxygen with FiO2 0.24 and was in room air at 31 hours post MIST for surfactant. Intubation and mechanical ventilation were avoided. At the fourth month follow-up, the infant is thriving well and his developmental milestones are appropriate.

Case 2

This 35 weeks/2.115 kg male neonate was delivered by emergency caesarian section and was initially on nasal CPAP from 30 minutes of life. Worsening of the distress warranted surfactant therapy by the fifth hour of life and curosurf 200 mg/kg was administered by MIST using a feeding tube and baby improved without the need for intubation and ventilation subsequently. Soon after instillation of curosurf baby had dropped saturation to 70s which had recovered spontaneously on continuation of nasal CPAP within three minutes. This vigorous baby was primed with intravenous morphine 50 micrograms/kg half an hour before the procedure.

Case 3

This 28 weeks/1.18 kg female neonate was delivered by emergency caesarian section to a G2 P1 L1 mother with pre-eclampsia who had received only a single dose of betamethasone before delivery of the baby. Despite early initiation of nasal bubble CPAP distress worsened with increasing oxygen requirements that warranted surfactant administration by nine hours of life. Curosurf 240 mg was administered by MIST using 5 F feeding tube that was easily guided across the glottis by finger guidance alone. Although this baby needed escalation of respiratory support due to persistent pulmonary hypertension and air leak syndrome that required draining bilateral pneumothoraces and high frequency oscillation for 48 hours, eventually baby made an uneventful recovery.

## Discussion

MIST has been in vogue in western neonatal units since 2010 and currently majority of neonatologists in Europe have adapted this as a routine in their neonatal units as revealed in an European survey [12]. In this survey it has been reported that the number of neonatal units practising less invasive surfactant administration (LISA) or MIST has shown a rapid growth from less than 10% in 2010 to 52% in 2015-2016 [12]. There are four ways of surfactant administration without intubation that has been reported in the literature [6]. These are: a) tracheal instillation using the thin flexible feeding tube assisted by Magill forceps or using semirigid vascular catheter or angiocaths, b) pharyngeal instillation of surfactant, c) by nebulizing surfactant, and d) using a laryngeal mask airway. Out of these four techniques, the first is widely practiced and c and d have some patronage. Intra-pharyngeal surfactant instillation technique was found to be an ineffective way [6].

The superiority of MIST over INSURE technique has been convincingly proven in a recent meta-analysis, MIST resulting in a 32.3% reduction of need for mechanical ventilation in the first 72 hours of life as compared to INSURE [11]. A significant 34.4% reduction in the risk of bronchopulmonary dysplasia with the use of thin catheter compared to INSURE has been reported (RR = 0.656; 95% CI = 0.375-1.149; P = 0.141), although results failed to reach statistical significance [11].

The adverse events noted during MIST have been bradycardia, desaturations, apnea and tracheal reflux of surfactant but these are transient and self-limiting in nature in majority of cases [6]. Our babies too had brief less than 30 seconds of bradycardia and desaturations following MIST but neither required intubation or escalation of respiratory support 48 hours post MIST. Premedications that have been considered are narcotic agents, midazolam, oral sucrose and intravenous propofol [6, 13]. In a Netherlands study of 38 neonates receiving MIST, 23 were sedated with propofol before the procedure whereas 15 were not and it was found that sedation resulted in increased need for nasal intermittent positive pressure ventilation and intubation and MIST failure rates were higher including failure to catheterize the trachea to administer surfactant [13]. Fifty-two percent of European neonatologists in a survey were not using sedatives for MIST [12].

Surfactant loss (efflux out of trachea post MIST) has been a concern and up to 11% loss of surfactant has been found with catheter instillation of surfactant in studies [14,15]. The loss was found to be higher with higher size catheters and administration of air boluses after surfactant instillation through catheter was found to minimize the loss especially the dead space volume of the catheter [15].

Our initial experience with MIST in our neonatal unit has been a satisfactory and rewarding experience and we feel this technique can be learnt with proper guidance and easily adapted in everyday practice to the benefit of our neonates.

## Conclusions

MIST is a skill that shall be mastered and implemented in the everyday clinical practice of neonatologists in this Gulf country and we believe that this novel technique will become the norm for surfactant administration in neonates.
